# Non contiguous-finished genome sequence and description of *Peptoniphilus senegalensis* sp. nov.

**DOI:** 10.4056/sigs.3366764

**Published:** 2013-02-22

**Authors:** Ajay Kumar Mishra, Jean-Christophe Lagier, Thi-Tien Nguyen, Didier Raoult, Pierre-Edouard Fournier

**Affiliations:** 1 Aix-Marseille Université, Faculté de médecine, Marseille, France

**Keywords:** *Peptoniphilus senegalensis*, genome

## Abstract

*Peptoniphilus senegalensis* strain JC140^T^ sp. nov., is the type strain of *P. senegalensis* sp. nov., a new species within the genus *Peptoniphilus*. This strain, whose genome is described here, was isolated from the fecal flora of a healthy patient. *P. senegalensis* strain JC140^T^ is an obligate Gram-positive anaerobic coccus. Here we describe the features of this organism, together with the complete genome sequence and annotation. The 1,840,641 bp long genome (1 chromosome but no plasmid) exhibits a G+C content of 32.2% and contains 1,744 protein-coding and 23 RNA genes, including 3 rRNA genes.

## Introduction

*Peptoniphilus senegalensis* strain JC140^T^ (= CSUR P154 = DSM 25694), is the type strain of *P. senegalensis* sp. nov. This bacterium is a Gram-positive, anaerobic, non spore-forming and indole positive coccus that was isolated from the stool of a healthy Senegalese patient as part of a “culturomics” study aiming at cultivating individually all species within human feces [1].

Since 1995 and the first sequencing of a bacterial genome, that of *Haemophilus influenzae*, more than 3,000 bacterial genomes have been sequenced [2]. This was made possible by technical improvements as well as increased interest in having access to the complete genetic information encoded by bacteria. In the meantime however, biological tools for defining new bacterial species have not evolved and DNA-DNA hybridization is still considered the gold standard [3] for determining sequence similarity despite its drawbacks, and the taxonomic revolution that has resulted from the comparison of 16S rDNA sequences [4]. Recently, we proposed to use a polyphasic approach to describe new bacterial taxa including their genome sequence, MALDI-TOF spectrum and main phenotypic characteristics (e.g., habitat, Gram staining, culture and metabolic characteristics, and when applicable, pathogenicity) [5-16].

Here we present a summary classification and a set of features for *P. senegalensis* sp. nov. strain JC140^T^ (= CSUR P154 = DSM 25694), together with the description of the complete genomic sequencing and annotation. These characteristics support the circumscription of the species *P. senegalensis*.

Gram-positive anaerobic cocci (GPAC) are part of the commensal flora in humans and animals and are also commonly associated with a variety of human infections [17], including vaginal discharges, ovarian, peritoneal, sacral and lacrymal gland abscesses [18]. In wide surveys of anaerobic infections, GPAC represent about 25 to 30% of all anaerobic isolates [19]. Extensive taxonomic changes have occurred among this group of bacteria, especially in clinically important genera such as *Peptostreptococcus* (Kluyver and van Niel 1936) [20], which was divided into the genera *Peptoniphilus* (Ezaki *et al.* 2001), *Anaerococcus* (Ezaki *et al.* 2001) and *Gallicola* (Ezaki *et al.* 2001) [18]. Currently, the genus *Peptoniphilus* is comprised of eight species [21], including *P. asaccharolyticus* (Ezaki *et al.* 2001), *P. harei* (Ezaki *et al.* 2001), *P. indolicus* (Ezaki *et al.* 2001), *P. ivorii* (Ezaki *et al.* 2001), *P. lacrimalis* (Ezaki *et al.* 2001) [18], *P. gorbachii* (Song *et al.* 2010), *P. olsenii* (Song *et al.* 2010) [22] and *P. methioninivorax* (Rooney *et al.* 2011) [23]. In addition, we recently proposed the creation of the species *P. timonensis* sp. nov. that was also isolated from the stool from the same patient as *P. senegalensis* sp. nov [10]. Members of the genus *Peptoniphilus* produce butyrate, are non-saccharolytic and use peptone and amino acids as major energy sources.

## Classification and features

A stool sample was collected from a healthy 16-year-old male Senegalese volunteer patient living in Dielmo (rural village in the Guinean-Sudanian zone in Senegal), who was included in a research protocol. Written assent was obtained from this individual. No written consent was needed from his guardians for this study because he was older than 15 years (in accordance with the previous project approved by the Ministry of Health of Senegal and the assembled village population and as published elsewhere [24]. Both this study and the assent procedure were approved by the National Ethics Committee of Senegal (CNERS) and the Ethics Committee of the Institut Fédératif de Recherche IFR48, Faculty of Medicine, Marseille, France (agreement numbers 09-022 and 11-017). Several other new bacterial species were isolated from this specimen using various culture conditions, including the recently described *Alistipes senegalensis*, *Alistipes timonensis*, *Anaerococcus senegalensis*, *Bacillus timonensis*, *Clostridium senegalense*, *Peptoniphilus timonensis, Paenibacillus senegalensis, Herbaspirillum massiliense, Kurthia massiliensis, Brevibacterium senegalense, Aeromicrobium massiliense and Cellulomonas massiliensis* [5-16]. The fecal specimen was preserved at -80°C after collection and sent to Marseille. Strain JC140^T^ (Table 1) was isolated in May 2011 by anaerobic cultivation on 5% sheep blood-enriched Columbia agar (BioMerieux, Marcy l’Etoile, France) at 37°C, after 10 days of preincubation of the stool sample in an anaerobic blood culture bottle enriched with 5 ml of sterile sheep blood.

**Table 1:** Classification and general features of *Peptoniphilus senegalensis* strain JC140^T^ according to the MIGS recommendations [25]

**MIGS ID**	**Property**	**Term**	**Evidence code^a^**
	Current classification	Domain: *Bacteria*	TAS [26]
		Phylum *Firmicutes*	TAS [27-29]
		Class *Clostridia*	TAS [30,31]
		Order *Clostridiales*	TAS [32,33]
		Family *Incertae sedis* XI	TAS [32,33]
		Genus *Peptoniphilus*	TAS [18]
		Species *Peptoniphilus senegalensis*	IDA
		Type strain JC140^T^	IDA
	Gram stain	Positive	IDA
	Cell shape	Coccoid	IDA
	Motility	Nonmotile	IDA
	Sporulation	Sporulating	IDA
	Temperature range	Mesophile	IDA
	Optimum temperature	37°C	IDA
MIGS-6.3	Salinity	Growth in BHI medium + 5% NaCl	IDA
MIGS-22	Oxygen requirement	Anaerobic	IDA
	Carbon source	Unknown	NAS
	Energy source	Unknown	NAS
MIGS-6	Habitat	Human gut	IDA
MIGS-15	Biotic relationship	Free living	IDA
MIGS-14	Pathogenicity Biosafety level Isolation	Unknown 2 Human feces	
MIGS-4	Geographic location	Senegal	IDA
MIGS-5	Sample collection time	September 2010	IDA
MIGS-4.1	Latitude Longitude	13.7167 -16.4167	IDA
MIGS-4.3	Depth	Surface	IDA
MIGS-4.4	Altitude	51 m above sea level	IDA

Evidence codes - IDA: Inferred from Direct Assay; TAS: Traceable Author Statement (i.e., a direct report exists in the literature); NAS: Non-traceable Author Statement (i.e., not directly observed for the living, isolated sample, but based on a generally accepted property for the species, or anecdotal evidence). These evidence codes are from the Gene Ontology project [34]. If the evidence is IDA, then the property was directly observed for a live isolate by one of the authors or an expert mentioned in the acknowledgements.

**Figure 1:**
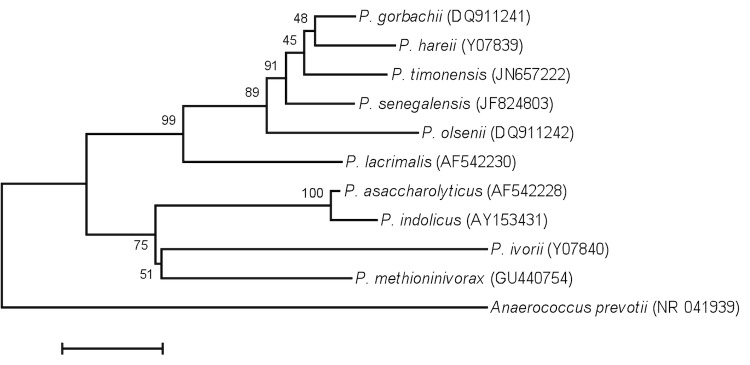
Phylogenetic tree highlighting the position of *Peptoniphilus senegalensis* strain JC140^T^ relative to other type strains within the *Peptoniphilus* genus. GenBank accession numbers are indicated in parentheses. Sequences were aligned using CLUSTALW, and phylogenetic inferences obtained using the maximum-likelihood method within the MEGA software. Numbers at the nodes are percentages of bootstrap values obtained by repeating the analysis 500 times to generate a majority consensus tree. *Anaerococcus prevotii* was used as outgroup. The scale bar represents a 2% nucleotide sequence divergence.

This strain exhibited a nucleotide sequence similarity with validly published *Peptoniphilus* species, ranging from 88.29% with *P. ivorii* (Ezaki *et al.* 2001) to 97.40% with *P. gorbachii* (Song *et al.* 2010) (Figure 1), values lower than the 98.7% 16S rRNA gene sequence threshold recommended by Stackebrandt and Ebers to delineate a new species without performing DNA-DNA hybridization [4]. In addition, strain JC140^T^ exhibited a 97.18% similarity with *P. timonensis* (GenBank accession number JN657222).

**Figure 2:**
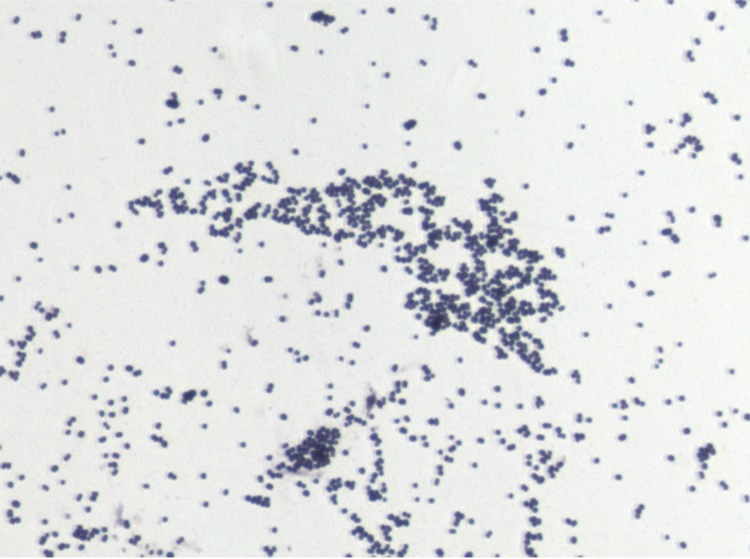
Gram staining of *P. senegalensis* strain JC140^T^

**Figure 3:**
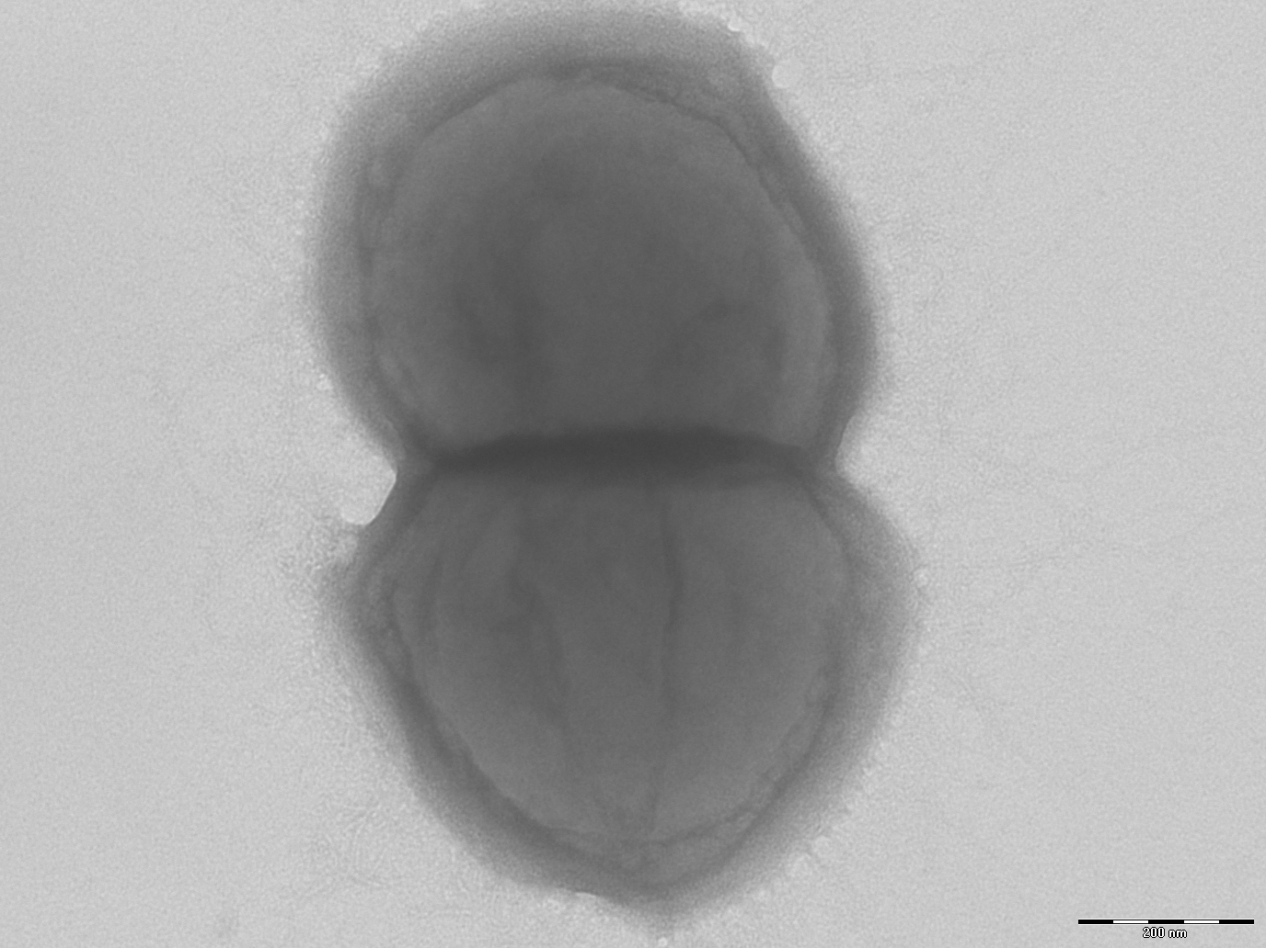
Transmission electron microscopy of *P. senegalensis* strain JC140^T^, using a Morgani 268D (Philips) at an operating voltage of 60kV.The scale bar represents 200 nm.

Different growth temperatures (25, 30, 37, 45°C) were tested; no growth occurred at 25°C or 45°C. Growth was observed between 30 and 37°C, but optimal growth was obtained at 37°C after 48 hr of inoculation. Colonies were 0.5 mm in diameter on blood-enriched Columbia agar and Brain Heart Infusion (BHI) agar. Growth of the strain was tested in anaerobic and microaerophilic conditions using GENbag anaer and GENbag microaer systems, respectively (BioMerieux), and under aerobic conditions, with or without 5% CO_2_. Growth was achieved only anaerobically. Gram staining showed Gram-positive cocci (Figure 2). A motility test was negative. Cells grown on agar have a mean diameter of 0.65 µm and are mostly grouped in pairs, short chains or small clumps (Figure 3).

Strain JC140^T^ exhibited a catalase activity but no oxidase activity. Using the API Rapid ID 32A system, positive reactions were observed for arginine arylimidase, tyrosine arylamidase, histidine arylamidase and indole production. Weak reactions were observed for leucyl glycine arylamidase and glycine arylamidase. All other assays were negative. *P. senegalensis* is susceptible to penicillin G, amoxicillin + clavulanic acid, imipeneme, vancomycin, clindamycin and metronidazole. By comparison with other phylogenetically closely related *Peptoniphilus* species, *P. senegalensis* differed in leucine arylamidase, phenylalanine arylamidase and serine arylamidase activities with *P. gorbachii* [22], in tyrosine arylamidase activity with *P. harei* [18] and in α-galactosidase, serine arylamidase, leucine arylamidase, phenylalanine arylamidase, glycine arylamidase and glycine arylamidase activities with *P. timonensis* [10].

**Figure 4:**
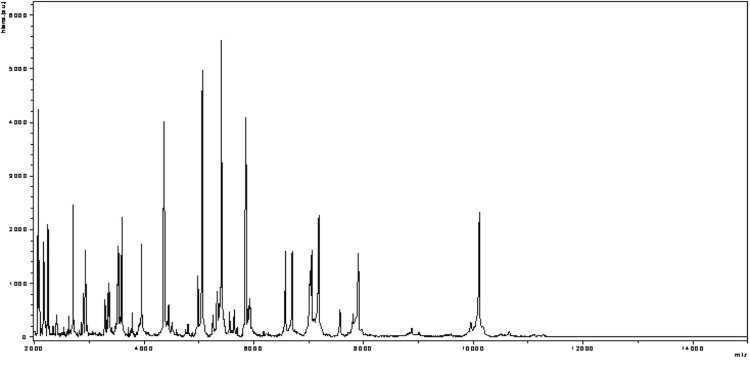
Reference mass spectrum from *P. senegalensis* strain JC140^T^. Spectra from 12 individual colonies were compared and a reference spectrum was generated.

Matrix-assisted laser-desorption/ionization time-of-flight (MALDI-TOF) MS protein analysis was carried out as previously described [35]. Briefly, a pipette tip was used to pick one isolated bacterial colony from a culture agar plate and spread it as a thin film on a MTP 384 MALDI-TOF target plate (Bruker Daltonics, Germany). Twelve distinct deposits were done for strain JC140^T^ from twelve isolated colonies. Each smear was overlaid with 2 µL of matrix solution (saturated solution of alpha-cyano-4-hydroxycinnamic acid) in 50% acetonitrile, 2.5% tri-fluoracetic acid, and allowed to dry for five minutes. Measurements were performed with a Microflex spectrometer (Bruker). Spectra were recorded in the positive linear mode for the mass range of 2,000 to 20,000 Da (parameter settings: ion source 1 (ISI), 20kV; IS2, 18.5 kV; lens, 7 kV). A spectrum was obtained after 675 shots at a variable laser power. The time of acquisition was between 30 seconds and 1 minute per spot. The twelve JC140^T^ spectra were imported into the MALDI BioTyper software (version 2.0, Bruker) and analyzed by standard pattern matching (with default parameter settings) against the main spectra of 3,769 bacteria, including spectra from 8 validly published *Peptoniphilus* species that were used as reference data in the BioTyper database (updated March 15^th^, 2012). The method of identification includes the m/z from 3,000 to 15,000 Da. For every spectrum, 100 peaks at most were taken into account and compared with the spectra in database. A score enabled the presumptive identification and discrimination of the tested species from those in a database: a score ≥ 2 with a validly published species enabled the identification at the species level; a score ≥ 1.7 but < 2 enabled the identification at the genus level; and a score < 1.7 did not enable an identification. For strain JC140^T^, the obtained score was 1.4, thus suggesting that our isolate was not a member of a known species. We incremented our database with the spectrum from strain JC140^T^ (Figure 4).

## Genome sequencing information

### Genome project history

The organism was selected for sequencing on the basis of its phylogenetic position and 16S rRNA similarity to other members of the genus *Peptoniphilus*, and is part of a “culturomics” study of the human digestive flora aiming at isolating all bacterial species within human feces. It was the ninth genome of a *Peptoniphilus* species and the first genome of *Peptoniphilus senegalensis* sp. nov. The genome was deposited in Genbank under accession number CAEL00000000 consists of 77 contigs. Table 2 shows the project information and its association with MIGS version 2.0 compliance [36].

**Table 2:** Project information

**MIGS ID**	**Property**	**Term**
MIGS-31	Finishing quality	High-quality draft
MIGS-28	Libraries used	One 454 paired end 3-kb library
MIGS-29	Sequencing platforms	454 GS FLX Titanium
MIGS-31.2	Fold coverage	45.38
MIGS-30	Assemblers	Newbler version 2.5.3
MIGS-32	Gene calling method	Prodigal
	Genbank ID	CAEL00000000
	Genbank Date of Release	November 19, 2012
MIGS-13	Project relevance	Study of the human gut microbiome

### Growth conditions and DNA isolation

*P. senegalensis* strain JC140^T^ (= CSUR P154 = DSM 25694), was grown anaerobically on 5% sheep blood-enriched Columbia agar at 37°C. Four petri dishes were spread and resuspended in 3×100µl of G2 buffer (EZ1 DNA Tissue kit, Qiagen). A first mechanical lysis was performed by glass powder on the Fastprep-24 device (Sample Preparation system, MP Biomedicals, USA) during 2×20 seconds. DNA was then treated with 2.5 µg/µL lysozyme (30 minutes at 37°C) and extracted through the BioRobot EZ1 Advanced XL (Qiagen). The DNA was then concentrated and purified on a Qiamp kit (Qiagen). The yield and the concentration was measured by the Quant-it Picogreen kit (Invitrogen) on a Genios_Tecan fluorometer at 42.2 ng/µl.

### Genome sequencing and assembly

A 3kb paired-end sequencing strategy (Roche, Meylan, France) was used. Five µg of DNA was mechanically fragmented on the Hydroshear device (Digilab, Holliston, MA, USA) with an enrichment size of 3-4kb for the construction of the paired-end library. The DNA fragmentation was visualized using an Agilent 2100 BioAnalyzer on a DNA labchip 7500 to yield an optimal size of 3.962kb. The library was constructed according to the 454 Titanium paired-end protocol (Roche). Circularization and nebulization were performed and generated a pattern with an optimum at 550 bp. Following PCR amplification through 15 cycles followed by double size selection, the single stranded paired-end library was quantified using the Quant-it Ribogreen kit (Invitrogen) on the Genios_Tecan fluorometer at 123pg/µL. The library concentration equivalence was calculated at 4.10E+08 molecules/µL. The library was held at -20°C until use.

The library was amplified with 1cpb in 4 emPCR reactions with the GS Titanium SV emPCR Kit (Lib-L) v2 (Roche). The yield of the emPCR was 9.13%, in the 5 – 20% range recommended by the Roche procedure. A total of 790,000 beads were loaded onto a one quarter region of a PTP Picotiterplate (PTP Kit 70×75, Roche) and pyrosequenced using the GS Titanium Sequencing Kit XLR70 and GS FLX Titanium sequencer (Roche). The run was performed overnight and then analyzed on the cluster through the gsRunBrowser and Newbler assembler (Roche). A total of 334,687 passed filter wells generated 83.5Mb with a length average of 249bp. The passed filter sequences were assembled using Newbler with 90% identity and 40 bp as overlap. The final assembly identified 39 contigs (>1500bp) arranged in 4 scaffolds and generated a genome size of 1.84Mb.

### Genome annotation

Open Reading Frames (ORFs) were predicted using Prodigal [37] with default parameters, but the predicted ORFs were excluded if they spanned a sequencing gap region. The predicted bacterial protein sequences were searched against the GenBank database [38] and the Clusters of Orthologous Groups (COG) databases using BLASTP. The tRNAScanSE tool [39] was used to find tRNA genes, whereas ribosomal RNAs were found by using RNAmmer [40] and BLASTN against the GenBank database. Signal peptides and numbers of transmembrane helices were predicted using SignalP [41] and TMHMM [42] respectively. ORFans were identified if their BLASTP *E*-value was lower than 1e-03 for an alignment length greater than 80 amino acids. If alignment lengths were smaller than 80 amino acids, we used an *E*-value of 1e-05. Such parameter thresholds have already been used in previous works to define ORFans.

To estimate the mean level of nucleotide sequence similarity at the genome level between *P. senegalensis* strain JC140^T^, *P. harei* strain ACS-146-V-Sch2b (GenBank accession number AENP00000000), *P. indolicus* strain ATCC29427 (AGBB00000000), and *P. timonensis* strain JC401^T^ (CAHE00000000), we compared the ORFs only using BLASTN and the following parameters: a query coverage of > 70% and a minimum nucleotide length of 100 bp.

Artemis [43] was used for data management and DNA Plotter [44] was used for visualization of genomic features. The Mauve alignment tool was used for multiple genomic sequence alignment and visualization [45]

## Genome properties

**Figure 5:**
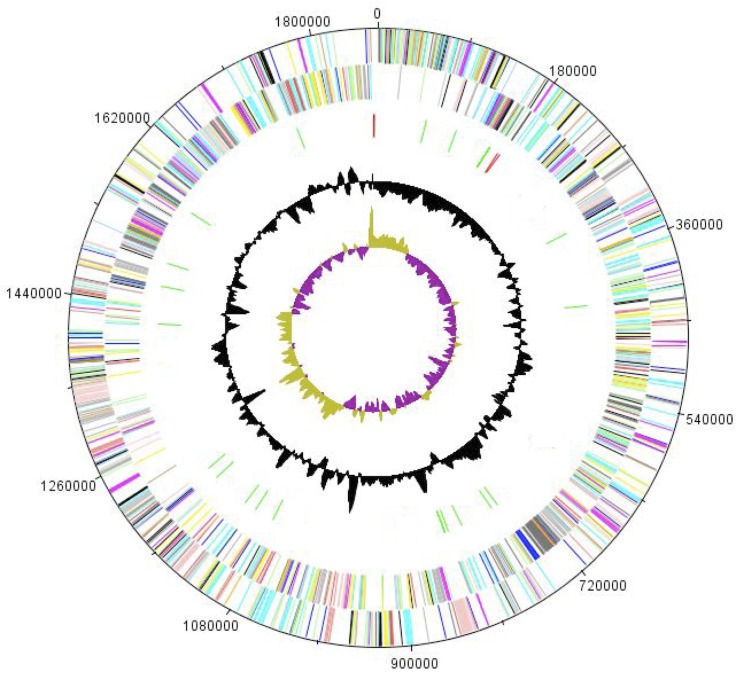
Graphical circular map of the chromosome. From the outside to the inside: genes on the forward strand (colored by COG categories), genes on the reverse strand (colored by COG categories), RNA genes (tRNAs green, rRNAs red), G+C content and GC skew.

The genome of *P. senegalensis* sp. nov. strain JC140^T^ is 1,840,641bp long (1 chromosome, but no plasmid) with a 32.2% G + C content (Figure 5 and Table 3). Of the 1,767 predicted genes, 1,744 were protein-coding genes, and 23 were RNAs, including a complete rRNA operon and 20 tRNAs. A total of 1,422 genes (80.47%) were assigned a putative function. A total of 86 genes were identified as ORFans (4.9%). The remaining genes were annotated as hypothetical proteins. The distribution of genes into COGs functional categories is presented in Table 4. The properties and the statistics of the genome are summarized in Tables 3 and 4.

**Table 3:** Nucleotide content and gene count levels of the genome

**Attribute**	**Value**	**% of total^a^**
Genome size (bp)	1,840,641	
DNA coding region (bp)	1,679,151	91.22
DNA G+C content (bp)	592,686	32.2
Number of replicons	1	
Extrachromosomal elements	0	
Total genes	1,767	100
RNA genes	23	1.30
rRNA operons	1	
Protein-coding genes	1,744	98.69
Genes with function prediction	1,422	80.47
Genes assigned to COGs	1,312	74.25
Genes with peptide signals	105	5.94
Genes with transmembrane helices	486	27.50
CRISPR repeats	0	

^a^ The total is based on either the size of the genome in base pairs or the total number of protein coding genes in the annotated genome

**Table 4:** Number of genes associated with the 25 general COG functional categories

**Code**	**Value**	**%age**^a^	**Description**
J	141	8.08	Translation
A	0	0	RNA processing and modification
K	102	5.85	Transcription
L	106	6.08	Replication, recombination and repair
B	1	0.06	Chromatin structure and dynamics
D	19	1.09	Cell cycle control, mitosis and meiosis
Y	0	0	Nuclear structure
V	62	3.56	Defense mechanisms
T	56	3.21	Signal transduction mechanisms
M	57	3.27	Cell wall/membrane biogenesis
N	4	0.23	Cell motility
Z	0	0	Cytoskeleton
W	0	0	Extracellular structures
U	22	1.26	Intracellular trafficking and secretion
O	58	3.33	Posttranslational modification, protein turnover, chaperones
C	92	5.28	Energy production and conversion
G	41	2.35	Carbohydrate transport and metabolism
E	115	6.59	Amino acid transport and metabolism
F	56	3.21	Nucleotide transport and metabolism
H	46	2.64	Coenzyme transport and metabolism
I	40	2.29	Lipid transport and metabolism
P	81	4.64	Inorganic ion transport and metabolism
Q	22	1.26	Secondary metabolites biosynthesis, transport and catabolism
R	179	10.26	General function prediction only
S	122	7.0	Function unknown
-	432	24.77	Not in COGs

^a^ The total is based on the total number of protein coding genes in the annotated genome.

## Comparison with other *Peptoniphilus* species *genome*

**Figure 6:**
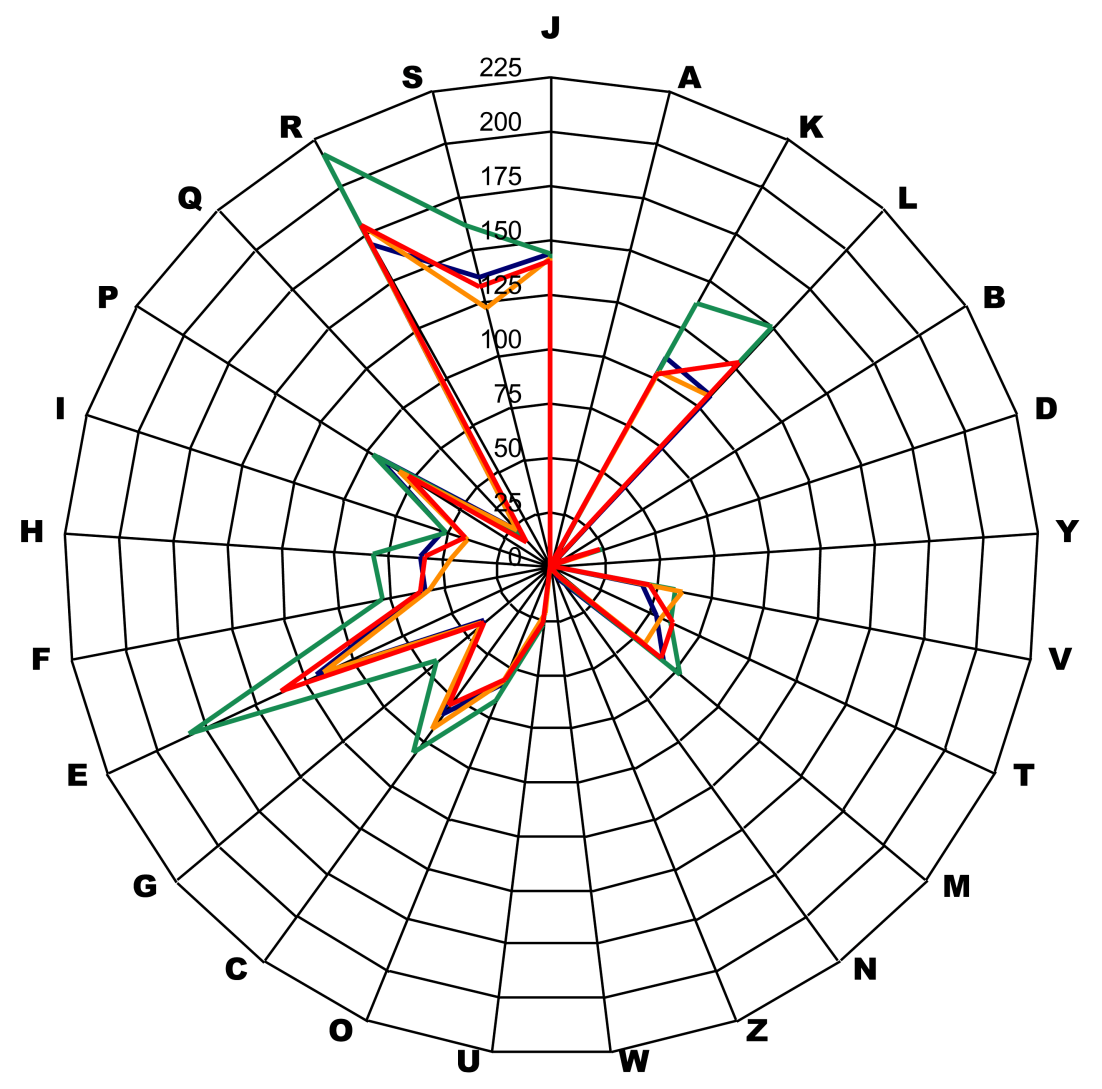
Gene distribution in COG functional categories in *P. senegalensis* (orange), *P. harei* (blue), *P. timonensis* (red) and *P. indolicus* (green) chromosomes.

Here, we compared the genome sequence of *P. senegalensis* strain JC140^T^ with those of *P. harei* strain ACS-146-V-Sch2b, *P. indolicus* strain ATCC 29427 and *P. timonensis* strain JC401^T^. The draft genome sequence of *P. senegalensis* is larger than *P. timonensis* and *P. harei* (1.84, 1.76 and 1.83 Mb, respectively), but smaller than *P. indolicus* (2.10 Mb). The G+C content of *P. senegalensis* is larger than *P. timonensis* (32.20 and 30.70%, respectively) but smaller than *P. indolicus* and *P. harei* (32.29 and 34.44%, respectively). Additionally, *P. senegalensis* has more predicted genes than *P. harei* (1,767 and 1,724, respectively), but fewer than *P. timonensis* and *P. indolicus* (1,922 and 2,269, respectively). The distribution of genes into COG categories was not entirely similar in all the three compared genomes (Figure 6). In addition, *P. senegalensis* shared a mean genome sequence similarity of 85.75% (range 70.12-100%), 84.75% (70.67-100%) and 85.60% (70.52-100%) with *P. harei*, *P. indolicus* and *P. timonensis*, respectively.

## Conclusion

On the basis of phenotypic, phylogenetic and genomic analyses, we formally propose the creation of *Peptoniphilus senegalensis* sp. nov. which contains the strain JC140^T^ as the sole member. This strain has been found in Senegal.

## Description of *Peptoniphilus senegalensis* sp. nov.

*Peptoniphilus senegalensis* (se.ne.gal.e’n.sis. L. gen. masc. n. *senegalensis*, pertaining to Senegal, the country where the specimen in which strain JC140^T^ was obtained).

Colonies are 0.5 mm in diameter on blood-enriched Columbia agar. Cells are coccoid with a mean diameter of 0.65 *μ*m, occurring mostly in pairs, short chains or small clumps. Optimal growth is achieved anaerobically. No growth is observed in aerobic or microaerophilic conditions. Growth occurs between 30 and 37°C, with optimal growth observed at 37°C on blood-enriched Columbia agar. Cells stain Gram-positive, are non endospore-forming, and non-motile. Cells are positive for catalase activity and indole production. Arginine arylamidase, tyrosine arylamidase, histidine arylamidase, leucyl glycine arylamidase and glycine arylamidase activities are present. *P. senegalensis* is susceptible to penicillin G, amoxicillin + clavulanic acid, imipeneme, vancomycin, clindamycin and metronidazole. The G+C content of the genome is 32.20%. The 16S rRNA and genome sequences are deposited in GenBank under accession numbers JN824803 and CAEL00000000, respectively. The type strain JC140^T^ (= CSUR P154 = DSM 25694) was isolated from the fecal flora of a healthy patient in Senegal.
